# Empowering Health Professionals with Digital Skills to Improve Patient Care and Daily Workflows

**DOI:** 10.3390/healthcare13030329

**Published:** 2025-02-05

**Authors:** Joao C. Ferreira, Luis B. Elvas, Ricardo Correia, Miguel Mascarenhas

**Affiliations:** 1 Faculty of Logistics, Molde University College, NO-6410 Molde, Norway; joao.carlos.ferreira@iscte-iul.pt (J.C.F.); luis.elvas@iscte-iul.pt (L.B.E.); 2 Instituto Universitário de Lisboa ISCTE—IUL, 1649-026 Lisboa, Portugal; 3 BioGHP, 1000-260 Lisboa, Portugal; ricardo@bioghp.com; 4 Department of Community Medicine, Information and Health Decision Sciences (MEDCIDS), Faculty of Medicine, University of Porto, 4099-002 Porto, Portugal

**Keywords:** digital health, healthcare education, digital proficiency, workflow optimisation, patient-centred care

## Abstract

The increasing digitalisation of healthcare has created a pressing need for health professionals to develop robust digital skills. This paper explores the imperative of equipping health professionals with the necessary digital proficiency to enhance their daily workflows and improve patient care. The expanding use of digital technologies, including electronic health records, telehealth, and artificial intelligence, has transformed the healthcare landscape. However, the adoption of these technologies has been hindered by barriers, such as a lack of interoperability and hesitancy among healthcare providers. To address these challenges, this paper argues that digital skill development must be a core component of healthcare education and professional training. Medical schools and healthcare organisations must prioritise the integration of digital health curricula and continuous learning opportunities to ensure that the next generation of healthcare providers is well-equipped to navigate the digital healthcare ecosystem. Additionally, this paper highlights the importance of fostering a culture of digital innovation and collaboration within healthcare settings. By empowering health professionals to actively participate in the development and testing of new digital health applications, the industry can unlock the full potential of digital technologies to enhance daily workflows and improve patient outcomes.

## 1. Introduction

In the rapidly evolving digital landscape, the healthcare sector is undergoing a profound transformation. The integration of technology into every facet of healthcare delivery demands that health professionals possess a robust set of digital skills to navigate this new terrain effectively, thus underlining the urgent need for educational initiatives that encompass both technical and clinical competencies to ensure providers can leverage digital tools to optimise patient care [1,2,3].

The healthcare sector is undergoing a profound transformation driven by the rapid advancement and integration of digital technologies. Electronic health records, telehealth platforms, data analytics tools, and myriad other digital solutions are revolutionising how healthcare is delivered, managed, and experienced. This wave of digitisation presents both immense opportunities and challenges for health professionals. As healthcare increasingly relies on these technologies, it is imperative that professionals engage in continuous learning and development to stay proficient, ultimately enhancing the overall quality of patient care and operational efficiency [2].

The incorporation of digital technologies into all aspects of healthcare delivery has required health professionals to have a strong foundation in digital skills in order to successfully navigate this new environment.

These digital skills encompass a wide range of capabilities, including proficiency in using electronic health records, navigating telehealth platforms, analysing health data, utilising digital communication tools, and staying abreast of emerging technologies. This comprehensive skill set not only enhances individual performance but also fosters a collaborative environment that ultimately leads to improved health outcomes and greater efficiency across healthcare systems [4]. Data-driven insights can also lead to the identification of patterns that enhance preventive care strategies and reduce medication errors, thereby creating a safer and more effective healthcare environment for patients [2].

In an era where patient information is increasingly digitised and healthcare processes are becoming more reliant on technology, these digital skills are no longer optional but essential for health professionals to deliver high-quality, efficient care. The ability to seamlessly leverage technology not only enhances efficiency and productivity but also directly impacts the quality of patient care and outcomes.

To thrive in this evolving landscape, it is imperative for healthcare workers to possess the necessary digital skills to navigate the complexities of modern healthcare delivery effectively. This paper aims to explore the critical importance of teaching digital skills to health professionals, focusing on how these skills can optimise daily workflows and ultimately contribute to a more efficient, patient-centred, and innovative healthcare system. Particular instances of digital skills and how they affect different facets of healthcare delivery will be examined, along with tactics that can be used to give health professionals these crucial skills.

### 1.1. Digital Skills: The Cornerstone of Modern Healthcare

Proficiency with electronic health records, telehealth platform navigation, health data analysis, analysing digital communication tools, and keeping up with emerging technologies are just a few of the many competencies that make up digital skills.

These competencies are now necessary for health professionals to provide high-quality, effective care in a time when patient data is becoming more digitalised and healthcare procedures are more and more dependent on technology [2,4].

The importance of digital skills in healthcare has been underscored by the Pan American Health Organization’s Eight Guiding Principles of Digital Transformation, which highlight the strategic actions required for advancing digital health, including universal connectivity and inclusive digital health through appropriate access, digital skills, and usability for the most vulnerable populations [1]. Data-driven insights can also lead to the identification of trends and disparities that inform public health initiatives, address social determinants of health, and enhance overall system effectiveness in a digital-first healthcare landscape [1,5].

### 1.2. Enhancing Daily Workflows

Digital skills enable health professionals to manage patient information seamlessly, facilitating quick access to medical histories, test results, and treatment plans, which translates to streamlined workflows, reduced administrative burdens, and more time devoted to direct patient care [2]. Improved communication and collaboration through digital tools, such as secure messaging platforms and video conferencing, foster the effective coordination of care plans and consultations among healthcare teams, leading to better patient outcomes. Data-driven decision-making empowered by the ability to analyse health data allows health professionals to not only inform diagnoses and treatment plans but also enhance preventive care strategies, thereby reducing the incidence of medical errors and optimising resource utilisation within healthcare systems. This capability ultimately positions healthcare providers to respond more effectively to patient needs while also aligning with broader efforts to improve the quality and accessibility of care through innovative digital solutions [1,2]. Moreover, the widespread integration of digital technologies, accelerated by recent global health challenges, highlights the necessity for ongoing training and support to ensure that all health professionals maintain a high level of digital competency, which is essential for navigating the complexities of modern healthcare environments and delivering optimal patient care.

### 1.3. The Role of Digital Skills in Modern Healthcare

Digital skills in the healthcare context encompass a broad range of capabilities that enable health professionals to effectively leverage technology and digital tools to enhance patient care and improve daily workflows. These skills include proficiency in using electronic health record systems to manage patient data, navigating telehealth platforms to deliver remote care, analysing health data to inform clinical decision-making, utilising secure digital communication tools to collaborate with healthcare teams, and staying up to date with emerging digital technologies in the field.

The significance of these digital skills cannot be overstated in the rapidly evolving healthcare landscape, where technology has become deeply integrated into every aspect of care delivery. As patient information becomes increasingly digitised and healthcare processes rely more heavily on digital tools, the possession of robust digital skills is no longer optional but essential for health professionals. Mastering these skills empowers clinicians to streamline administrative tasks, enhance communication and collaboration, make data-driven decisions, and ultimately devote more time to direct patient care. Furthermore, the widespread integration of digital technologies, accelerated by recent global health challenges, has underscored the critical importance of ongoing training and support to ensure all healthcare providers maintain a high level of digital competency to navigate the complexities of modern healthcare environments and deliver optimal patient-centred care.

### 1.4. Major Digital Skills in Healthcare Extracted from a Literature Review

#### 1.4.1. Electronic Health Records

Proficiency in using electronic health record systems is a critical digital skill for health professionals. EHRs serve as the central repository for patient data, allowing practitioners to quickly access comprehensive medical histories, laboratory results, medication records and other crucial information. Mastering EHR navigation and data entry enables the efficient documentation of clinical encounters, treatment plans, and care coordination. Furthermore, EHR competency empowers health professionals to leverage advanced functionalities, such as automated clinical decision support, predictive analytics, and population health management tools, to enhance the quality, safety, and cost-effectiveness of care delivery. Proficiency in using electronic health record systems is a critical digital skill for health professionals, as EHRs serve as the central repository for patient data, allowing practitioners to quickly access comprehensive medical histories, laboratory results, medication records and other crucial information, thus empowering health professionals to leverage advanced functionalities, such as automated clinical decision support and predictive analytics, to enhance the quality and cost-effectiveness of care delivery [2,6].

#### 1.4.2. Telehealth Skills

Proficiency in leveraging telehealth platforms and technologies to conduct virtual consultations, enable remote patient monitoring, and deliver healthcare services remotely is essential. This includes proficiency in operating video conferencing software, managing patient data and records securely within telehealth systems, and effectively engaging patients through digital channels to provide high-quality, accessible care from a distance. Mastering telehealth skills allows health professionals to extend their reach, improve patient convenience and satisfaction, and enhance care coordination, especially for vulnerable populations or those with limited access to in-person healthcare resources. Moreover, with a strong foundation for proactive health interventions and strategies that ultimately contribute to a more equitable healthcare environment, they are also able to leverage technology to close gaps in care for disadvantaged communities. This strong foundation for proactive health interventions and strategies ultimately contributes to a more equitable healthcare environment, leveraging technology to close gaps in care for disadvantaged communities, as evidenced by the promising results seen in the use of telemedicine and remote monitoring to improve access to healthcare in rural and underserved areas.

#### 1.4.3. Health Data Analysis

The ability to efficiently analyse health data empowers health professionals to make more informed decisions regarding diagnoses, treatment plans, and preventive care strategies. By leveraging data-driven insights, healthcare providers can gain a deeper understanding of patient trends, identify risk factors, and develop proactive interventions to improve individual and population health outcomes. This data-driven approach allows for personalised care, reduced medical errors, and optimised resource utilisation within the healthcare system. Mastering health data analysis skills is crucial for transforming raw data into actionable intelligence that can drive innovation. Furthermore, this mastery also improves the quality, safety, and cost-effectiveness of care delivery, thereby ensuring that healthcare professionals are not only equipped to meet current demands but are also prepared to embrace the future of healthcare innovations as they emerge [2,7,8]. This reinforces the imperative for continuous professional development and training in digital skills that align with evolving technological advancements and healthcare demands.

#### 1.4.4. Digital Communication Tools

The proficient use of secure messaging platforms, video conferencing software, and other digital collaboration tools enables effective communication and enhanced coordination among healthcare teams. These digital communication channels facilitate timely consultations, the exchange of critical patient information, and the development of coordinated care plans, ultimately leading to improved patient outcomes. By leveraging digital tools, healthcare professionals can seamlessly share updates, coordinate treatment strategies, and engage in real-time discussions, breaking down geographic barriers and fostering a more collaborative, patient-centric approach to patient care delivery. In addition, the healthcare system’s responsiveness is also refined, leading to the more effective management of population health and reduced disparities in access to patient care. Also, the implementation of predictive analytics can provide foresight into patient needs and streamline resource allocation across healthcare settings, ultimately improving care delivery efficiency and effectiveness [9].

#### 1.4.5. Emerging Technologies

Keeping pace with the rapid advancement of technology is crucial for health professionals. Staying informed about and adapting to new digital tools, innovations and platforms in the healthcare landscape is essential for delivering high-quality, efficient, and patient-centric care. This includes exploring and mastering the use of emerging technologies, such as artificial intelligence, machine learning, robotics, virtual and augmented reality, and digital therapeutics. By continuously enhancing their digital skills and embracing technological innovations, health professionals can leverage these tools to streamline workflows, improve clinical decision-making, enhance patient engagement, and drive meaningful progress in the field of healthcare. In doing so, they can contribute to the development of a more responsive, adaptive, and technologically advanced healthcare system that is better equipped to meet the evolving needs of patients and healthcare organisations alike, ultimately paving the way for enhanced patient experiences and improved population health outcome

Additionally, this document emphasises the importance of digital skills in the following contexts:

- Streamlining workflows and reducing administrative burdens through the efficient management of patient information and digital documentation processes.
- Enhancing communication and collaboration among healthcare teams through secure digital communication tools, enabling timely consultations, coordinated care planning, and improved information sharing.
- Empowering data-driven decision-making by leveraging the analysis of health data to inform diagnoses, treatment strategies, and preventive care initiatives, leading to more personalised and effective patient outcomes.
- Delivering enhanced patient care and improved health outcomes by leveraging digital technologies to optimise clinical workflows, facilitate remote patient monitoring and virtual consultations, and provide personalised, data-driven healthcare services.

How can the outlined digital skills optimise daily workflows, enhance patient care, and drive innovation in the healthcare field?

This strategic focus on developing digital competencies not only ensures that health professionals are equipped to handle the complexities of modern healthcare but also fosters a culture of continuous improvement and adaptability within healthcare systems, ultimately leading to enhanced patient experiences and outcomes across diverse populations [6] and settings [2,10].

## 2. Literature Review

The healthcare sector has been significantly impacted by the quick development of digital technologies, which has ushered in a new era of care delivery enabled by technology [11]. The way healthcare professionals communicate with patients, obtain medical information, and make clinical decisions has changed as a result of the integration of electronic health records, telehealth platforms, and artificial intelligence-powered decision support systems. For healthcare organisations, this digital transformation could result in increased productivity, better patient outcomes, and financial gains.

However, the adoption of these digital health technologies has been hampered by a number of obstacles, including a lack of interoperability, data security concerns, and reluctance among healthcare providers [12]. To overcome these challenges, health professionals must develop strong digital skills in order to effectively navigate the changing healthcare landscape.

The case for curricular change within educational institutions is well-documented. The current clinical workforce is not adequately trained or equipped to capitalise on the potential benefits of digital health technologies. Tuition in ethical, privacy, and security issues related to health information systems is also lacking, leaving healthcare providers ill-prepared to address the complex legal and ethical considerations that arise with the increased use of digital tools.

To address this gap, medical schools and healthcare organisations must prioritise the integration of digital health curricula and continuous learning opportunities. As highlighted by the Topol Review, all clinicians must have a basic understanding of the role of digital technologies in healthcare, along with practical training in using them to benefit patients.

Medical students and healthcare professionals must be equipped with a comprehensive set of digital competencies, including proficiency in data management, telemedicine, and the ethical use of artificial intelligence. By embedding these digital skills into professional training curricula and providing ongoing support and resources, healthcare organisations can empower their workforce to leverage digital technologies, enhance daily workflows, and improve patient care.

Recent research has emphasised compelling possibilities and unresolved challenges of digital health technologies [13]. The COVID-19 pandemic has accelerated the adoption of digital solutions, such as telemedicine, and has highlighted the importance of interoperability and digitalisation in healthcare [14]. Digital healthcare holds significant potential to improve patient outcomes, enhance efficiency, and generate economic benefits for healthcare organisations [15].

However, the widespread adoption of digital health technologies has been hindered by various barriers, including a lack of interoperability, resistance to change, and inadequate digital skills among healthcare professionals [11,15].

To address these challenges, medical schools and healthcare organisations must prioritise the integration of digital health curricula and continuous learning opportunities. According to the Topol Review, all clinicians need to be trained practically in using digital technologies to help patients and have a fundamental understanding of their role in healthcare.

Digital competencies need to be defined and embedded into professional training curricula, and support must be provided to healthcare professionals to create and test new digital health applications.

Moreover, the acceptance and utilisation of digital health technologies by students and healthcare professionals is crucial for their successful integration into mainstream healthcare.

Furthermore, fostering a culture of digital innovation in healthcare settings is crucial. By empowering health professionals to develop and test new digital health tools, the industry can unlock the full potential of these technologies to streamline workflows and enhance patient-centred care.

### 2.1. Cultivating a Culture of Digital Innovation

Beyond the integration of digital health curricula, it is essential to foster a culture of digital innovation and collaboration within healthcare settings.

By empowering health professionals to actively participate in the development and testing of new digital health applications, the industry can unlock the full potential of these technologies to enhance daily workflows and improve patient outcomes.

Bridging the Digital Skills Gap in Healthcare, that is, to fully harness the transformative potential of digital health, it is imperative that healthcare professionals develop robust digital competencies, as the current healthcare workforce is not adequately trained or equipped to effectively leverage digital technologies to benefit patients.

A growing body of literature emphasises the need for comprehensive digital health education within healthcare curricula and continuous professional development programmes [3]. By empowering healthcare professionals with the necessary digital skills, organisations can streamline daily workflows, enhance decision-making, and ultimately provide more personalised and patient-centric care.

### 2.2. Integrating Digital Health into Healthcare Education and Training

The integration of digital health education into healthcare curricula and continuous professional development programmes is crucial to bridging the digital skills gap. Medical schools and healthcare organisations must prioritise the development of digital proficiency, including practical training in the use of digital health technologies, as well as ethical considerations and data privacy issues.

Recent studies have underscored the critical need to define and integrate digital health competencies into professional training curricula, ensuring that upcoming healthcare providers are adept at navigating the digital healthcare landscape and utilising digital tools to enhance patient care. Notable references are as follows:

1. “Digital Health Competencies in Medical School Education: A Scoping Review and Delphi Method Study”. This study identifies essential knowledge, skills and attitudes for a digital health curriculum in medical education, emphasising the importance of equipping future clinicians with competencies to effectively use digital health tools [3].
2. “Healthcare Professionals’ Digital Health Competence Profiles and Their Associated Factors”. This research explores the digital health competence profiles of healthcare professionals, highlighting the necessity for targeted training to ensure the effective utilisation of digital tools in clinical practice [16].
3. “Bridging the Educational Gap in Terms of Digital Competences Between Education and Healthcare Practice”. This article discusses the disparity between current educational offerings and the digital competencies required in healthcare practice, advocating for curriculum reforms to better prepare healthcare professionals [17].

The UK Topol Review emphasises the need for all clinicians to possess a fundamental understanding of digital technologies in healthcare, paired with practical training to harness these tools for patient benefit [18]. Integrating digital health proficiency into healthcare education is therefore essential for the preparation of future providers with the skills required to excel in the increasingly digital landscape of medicine [19].

## 3. The Transformative Potential of Digital Health

Rapid advancements in digital technologies have profoundly impacted the healthcare industry, ushering in a new era of digitally enabled care delivery [1].

Despite the potential for transformation of the industry, a number of obstacles have prevented the widespread use of these digital health technologies, such as a lack of interoperability, worries about data security, and reluctance on the part of healthcare providers [2]. Once again, health professionals must build strong digital competencies to successfully navigate the changing healthcare landscape in order to overcome these obstacles.

### 3.1. Empowering Health Professionals with Digital Skills

The case for curricular change within educational institutions is well-documented. The current clinical workforce is not adequately trained or equipped to capitalise on the potential benefits of digital health technologies.

Recent research has emphasised the compelling possibilities and unresolved challenges of digital health technologies [3]. The COVID-19 pandemic has accelerated the adoption of digital solutions, such as telemedicine, and has highlighted the importance of interoperability and digitalisation in healthcare [4]. Digital healthcare holds significant potential to improve patient outcomes, enhance efficiency, and generate economic benefits for healthcare organisations [5].

### 3.2. Bridging the Digital Skills Gap in Healthcare

To fully harness the transformative potential of digital health, it is imperative that healthcare professionals develop robust digital proficiency. The current healthcare workforce is not adequately trained or equipped to effectively leverage digital technologies to benefit patients.

### 3.3. Integrating Digital Health into Healthcare Education and Training

To close the digital skills gap, digital health education must be incorporated into healthcare curricula and ongoing professional development initiatives. The development of digital proficiency, which includes hands-on instruction in the use of digital health technologies, ethical considerations, and data privacy issues, must be given top priority in medical schools and healthcare institutions.

Recent studies have highlighted the importance of defining and embedding digital health competencies into professional training curricula [6]. This approach ensures that the next generation of healthcare providers is well-equipped to navigate the digital healthcare landscape and effectively use digital tools to enhance patient care.

### 3.4. Fostering a Culture of Digital Innovation in Healthcare

Beyond the integration of digital health education, healthcare organisations must also cultivate a culture of digital innovation and collaboration. By empowering healthcare professionals to actively participate in the development and testing of new digital health applications, the industry can unlock the full potential of digital technologies to streamline daily workflows and improve patient outcomes.

## 4. Digital Skills as a Catalyst for Innovation

Health professionals with strong digital skills are uniquely positioned to embrace and drive technological innovation in the healthcare sector. By possessing a deep understanding of digital tools, healthcare data, and emerging technologies, these professionals can identify opportunities to integrate new innovations into daily workflows and patient care processes. They can recognise the practical applications of technologies such as artificial intelligence, telemedicine, and digital therapeutics and spearhead the adoption and implementation of these transformative solutions within their organisations. With their digital expertise, they can collaborate effectively with technology teams, provide valuable user insights, and champion the development of customised digital health innovations that address the specific needs and challenges faced by healthcare providers and patients. This synergy between clinical knowledge and digital proficiency empowers health professionals to actively shape the future of healthcare, steering the industry towards a more technologically advanced, data-driven, and patient-centric model of care delivery. By leading the charge in digital health innovation, these professionals can unlock new avenues for improving clinical outcomes, enhancing operational efficiency, and driving meaningful progress in the field of healthcare. The capacity to leverage these digital tools not only enhances operational efficiency but also significantly contributes to bridging the gaps in patient care delivery, particularly in underserved communities, thereby promoting equitable health outcomes for all patients [3].

Health professionals with strong digital skills are uniquely positioned to embrace and leverage emerging technologies, such as artificial intelligence, telemedicine, and digital therapeutics, as they are able to deliver highly personalised and efficient healthcare.

For instance, AI-powered predictive analytics can provide valuable insights to inform personalised treatment plans, while telemedicine facilitates remote patient monitoring and virtual consultations, thereby improving access to care. Similarly, digital therapeutics can empower patients to actively manage their own health through personalised digital interventions. By harnessing the power of these emerging technologies, digitally competent health professionals can streamline operations, improve clinical decision-making, and ultimately provide more tailored, efficient, and accessible care to their patients. This strategic focus on leveraging digital innovations not only enhances the quality of care but also promotes greater equity in healthcare delivery, particularly for underserved communities.

### Challenges and Barriers

While the importance of digital skills is widely recognised, several challenges and barriers hinder their acquisition and implementation, as seen in Table 1.

Identifying major digital skills in healthcare involves analysing current and emerging trends in the industry, evaluating the needs of healthcare systems, and assessing the digital tools that improve clinical outcomes, efficiency, and patient care. Major digital skills were identified in Table 1. We chose to interview health professionals, conduct a scientific literature review, and review reports from consulting companies related to health.

To complement Table 1, we create an additional Table 2, with information about digital skills extracted from scientific papers identified in current work.

Proficiency in using electronic health record systems is a critical digital skill for health professionals. EHRs serve as the central repository for patient data, allowing practitioners to quickly access comprehensive medical histories, laboratory results, medication records, and other crucial information. Mastering EHR navigation and data entry facilitates the efficient documentation of clinical encounters, treatment plans, and patient care coordination. Furthermore, EHR competency empowers health professionals to leverage advanced functionalities, such as automated clinical decision support, predictive analytics, and population health management tools. The use of these tools enables the enhancement of the quality, safety, and cost-effectiveness of patient care delivery [2,6].

As for proficiency in leveraging telehealth platforms and technologies, these skills power the management of virtual consultations, enable remote patient monitoring, and deliver healthcare services remotely. This includes proficiency in operating video conferencing software, managing patient data and records securely within telehealth systems, and effectively engaging patients through digital channels to provide high-quality, accessible care from a distance. Therefore, mastering telehealth skills allows health professionals to extend their reach, improve patient convenience and satisfaction, and enhance care coordination, especially for vulnerable populations or those with limited access to in-person healthcare resources. This strong foundation for proactive health interventions and strategies ultimately contributes to a more equitable healthcare environment, leveraging technology to close gaps in care for disadvantaged communities, as evidenced by the promising results seen in the use of telemedicine and remote monitoring to improve access to healthcare in rural and underserved areas.

Regarding efficient health data analysis, this ability empowers health professionals to make more informed decisions regarding diagnoses, treatment plans, and preventive care strategies. By leveraging data-driven insights, healthcare providers can gain a deeper understanding of patient trends, identify risk factors, and develop proactive interventions to improve individual and population health outcomes. This data-driven approach allows for personalised patient care, reduced medical errors, and the optimised use of resources within the healthcare system. Mastering health data analysis skills is crucial for transforming raw data into actionable intelligence that can drive innovation and enhance the quality, safety, and cost-effectiveness of care delivery, thereby ensuring that healthcare professionals are not only equipped to meet current demands but are also prepared to embrace the future of healthcare innovations as they emerge [2,7,8], thereby reinforcing the imperative for continuous professional development and training in digital skills that align with evolving technological advancements and healthcare demands.

The proficient use of digital communication tools, namely, the use of secure messaging platforms, video conferencing software, and other digital collaboration tools, enables effective communication and enhanced coordination among healthcare teams. These digital communication channels facilitate timely consultations, the exchange of critical patient information, and the development of coordinated care plans, ultimately leading to improved patient outcomes. Furthermore, by leveraging digital tools, healthcare professionals can seamlessly share updates, coordinate treatment strategies, and engage in real-time discussions, breaking down geographic barriers and fostering a more collaborative, patient-centric approach to care delivery. These improvements enhance a healthcare system’s responsiveness, leading to the more effective management of population health and reduced disparities in access to care. In addition, the implementation of predictive analytics can also be significant, as it can provide foresight into patient needs and streamline resource allocation across healthcare settings, ultimately improving patient care delivery efficiency and effectiveness [9].

Regarding the various emerging technologies, there is a need to keep pace with the rapid advancement of technology, which is crucial for health professionals. Staying informed about and adapting to new digital tools, innovations, and platforms in the healthcare landscape is essential for delivering high-quality, efficient, and patient-centric care. This includes exploring and mastering the use of emerging technologies, such as artificial intelligence, machine learning, robotics, virtual and augmented reality, and digital therapeutics. By continuously enhancing their digital skills and embracing technological innovations, health professionals can leverage these tools to streamline workflows, improve clinical decision-making, enhance patient engagement, and drive meaningful progress in the field of healthcare. In doing so, they can contribute to the development of a more responsive, adaptive, and technologically advanced healthcare system that is better equipped to meet the evolving needs of patients and healthcare organisations alike, ultimately paving the way for enhanced patient experiences and improved population health outcomes.

This study was conducted within a Digital Health Master’s programme to identify the digital skills essential for healthcare professionals and evaluate the level of mastery across various professional roles in the healthcare ecosystem. The participants included 90 healthcare professionals representing a diverse range of roles, namely, 35 nurses, 33 physicians, eight pharmacists, seven radiologists, and seven administrative healthcare workers. Among the participants, 62 were women, and among all participants, 71 had two or more years of experience. Regarding their age, 25 contributors were younger than 30 years, and 25 were anywhere between 31 and 50 years old. Finally, there were nine participants who were older than 50 years.

The participants were asked to respond to open-ended, written questions designed to explore (1) the digital skills they perceive as necessary in their professional roles; (2) the digital tools and technologies they regularly use or require to perform their tasks effectively; and (3) challenges or gaps they face in acquiring or utilising digital competencies. This open-question format was chosen to allow respondents to articulate their needs and experiences freely, providing rich, qualitative data about the intersection of digital technology and their work environments. Within these questions, participants were asked to describe the specific tools they use in their daily practice, such as EHRs, telemedicine platforms, and diagnostic software. Moreover, the questions were aimed at uncovering areas in which professionals feel underprepared, such as data analytics, cybersecurity awareness, or interoperability standards. The main object of this study was to understand how the mastery of digital skills enhanced their ability to deliver patient care, collaborate with peers, and comply with evolving healthcare standards.

After the answers are analysed, this study aims to (1) identify common digital skill sets essential across healthcare roles; (2) highlight unique skill requirements for specific professions (e.g., radiologists vs. administrative workers); and (3) provide actionable insights for developing tailored digital health training programmes that address the gaps and promote mastery of critical digital competencies. Moreover, this study will contribute to understanding the digital transformation needs in healthcare and support the creation of education and training initiatives to prepare healthcare professionals for a digitally driven future.

**Figure 1:**
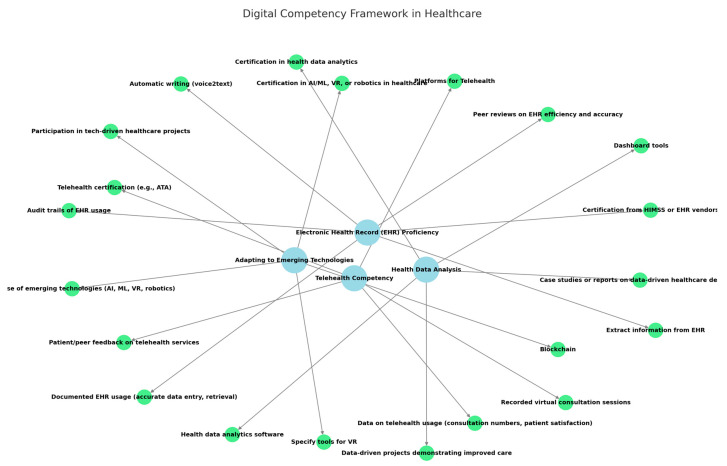
Major topics and relations from digital skills from health professionals’ answers.

To analyse the responses, a word frequency analysis was employed, which identified recurring terms and phrases. The process included the following steps:

- Data preprocessing: Responses were cleaned by removing stop words, standardising terminology (e.g., “EHR” and “electronic health record”), and correcting inconsistencies.
- Frequency analysis: A software tool ([e.g., NVivo, Python], https://lumivero.com/products/nvivo/, accessed on 6 December 2024) was used to quantify the occurrence of words and phrases.
- Thematic grouping: High-frequency terms were grouped into thematic categories, such as technical skills, data management, and digital communication, based on contextual relevance.
- Clustering and validation: Themes were clustered iteratively, combining automated analysis with manual review to ensure accuracy and alignment with the data. Independent reviewers validated the final clusters to enhance robustness.
- Result presentation: The clustered themes and their frequencies are summarised in Table 3 and Figure 1.

The 90 health professionals are categorised into three experience groups as follows:

- Less than two years (early career professionals): represents ~20% of participants (approximately 18 individuals); includes recent graduates and entry-level practitioners; and provides insights into how early career professionals adapt to digital transformation in healthcare.
- Two to five years (mid-level professionals): represents ~35% of participants (approximately 32 individuals) and includes professionals with intermediate experience who are likely to engage actively with digital tools and provide valuable insights into transitioning workflows.
- More than five years (experienced professionals): represents ~45% of participants (approximately 40 individuals); includes senior professionals with extensive clinical and administrative experience; and offers an in-depth understanding of long-term system integration and potential resistance to change.

Regarding their geographical origin, participants are mainly from the following countries:

- Portugal: ~40% (approximately 36 individuals), representing diverse healthcare settings such as urban hospitals, rural clinics, and specialised centres.
- Greece: ~30% (approximately 27 individuals), focusing on both public and private healthcare systems.
- Finland: ~30% (approximately 27 individuals), emphasising advanced digital healthcare practices and interoperability challenges.

Participants were selected based on the following criteria:

- Professional role: inclusion of diverse roles such as physicians, nurses, radiographers, IT specialists, and administrators to ensure a comprehensive perspective.
- Experience levels: balanced representation across early career, mid-level, and experienced professionals to capture a range of insights.
- Geographic diversity: targeted inclusion from Portugal, Greece, and Finland to explore regional variations in healthcare practices and digital transformation readiness.
- Familiarity with digital tools: preference for individuals with exposure to digital health systems, ensuring relevance to this study’s objectives.
- Healthcare setting: representation from different healthcare environments (e.g., urban vs. rural, public vs. private) to address varied challenges and opportunities.
- Voluntary participation: participants were recruited through professional networks and healthcare associations, ensuring willingness and availability for detailed feedback.

By combining these validation methods, healthcare professionals can effectively demonstrate their proficiency in digital skills and prove that they are well-equipped to navigate the ever-evolving technological landscape in healthcare.

However, while the importance of digital skills is widely recognised, several challenges and barriers hinder their acquisition and implementation, as seen in Table 4, which was created based on the 90 health professionals’ written statements.

To effectively integrate digital health solutions and enhance digital literacy among health professionals, the following strategies should be implemented (Table 5).

**Figure 2:**
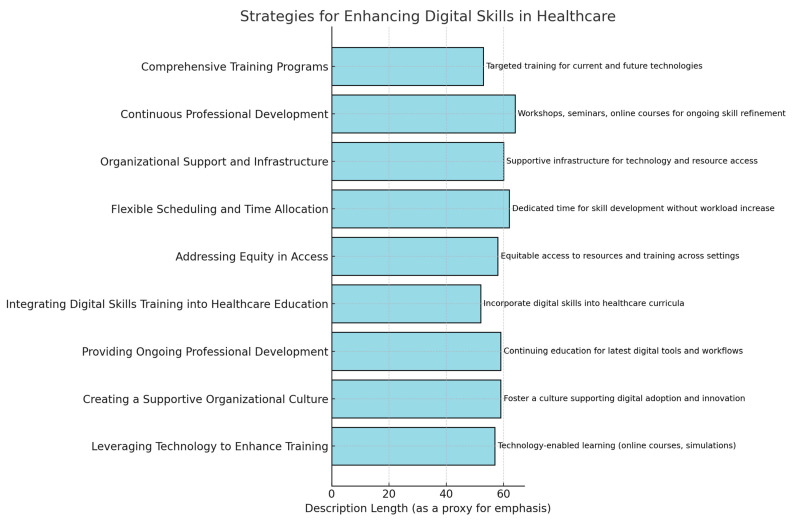
Strategies for enhancing digital skills in healthcare obtained from the written answers of health professionals.

To promote the adoption of digital skills among health professionals, several key strategies can be implemented, as seen in Table 6 and Figure 2, which were identified via a literature review.

By implementing these strategies and addressing the existing challenges, healthcare systems can empower health professionals to harness the full potential of digital skills, ultimately enhancing daily workflows, improving patient care, and driving innovation in the field of healthcare.

## 5. Major Findings from Our Questionnaire

The results of this study highlighted several key areas of focus in digital transformation within healthcare. These topics encompass both the opportunities and challenges associated with integrating digital technologies into healthcare workflows. A summary of the major topics identified is as follows:

- Digital transformation: the overarching process of integrating digital technologies into healthcare.
- Patient care: enhancing the quality and personalisation of care, leading to better outcomes.
- Artificial intelligence (AI): Leveraging AI for diagnostics, treatment planning, and data analysis.
- Telemedicine and remote monitoring: technologies that expand healthcare access and improve efficiency.
- Electronic health records (EHRs): central to data storage and accessibility.
- Interoperability: ensuring seamless integration and communication among healthcare systems.
- Operational efficiency: streamlining processes and reducing costs through digital tools.
- Privacy and security: addressing concerns about protecting sensitive patient data.
- Predictive analytics and big data: harnessing data insights to improve decision-making and patient care.

### 5.1. Cluster Analysis

- The clustering methodology revealed the most frequently mentioned terms and themes in student responses. These clusters were based on word frequency analysis and grouped into overarching themes. The key clusters are as follows:
- Digital skills: proficiency in EHRs, telemedicine, and AI tools.
- Challenges: concerns about privacy, digital literacy, and implementation costs.
- Benefits: improved patient outcomes, operational efficiency, and accessibility.
- Graphical representations (e.g., word clouds, bar charts) of these clusters will be added to enhance clarity and engagement for readers.

Therefore, this study identified the following significant benefits of digital transformation:

- Improved patient care and outcomes: personalised treatments, faster diagnoses, and enhanced care delivery.
- Increased efficiency: reduction in administrative burdens and streamlined workflows.
- Enhanced accessibility: telehealth solutions reaching underserved populations.
- Data-driven insights: improved clinical decision-making through analytics and predictive tools.

However, a couple of participants expressed several concerns about digital transformation as follows:

- Privacy and data security: fear of breaches and ethical misuse of patient data.
- Digital literacy gaps: the need for adequate training for healthcare professionals.
- Over-reliance on technology: concerns about reduced human oversight and empathy.

### 5.2. Comparison to the Previous Literature

**Figure 3:**
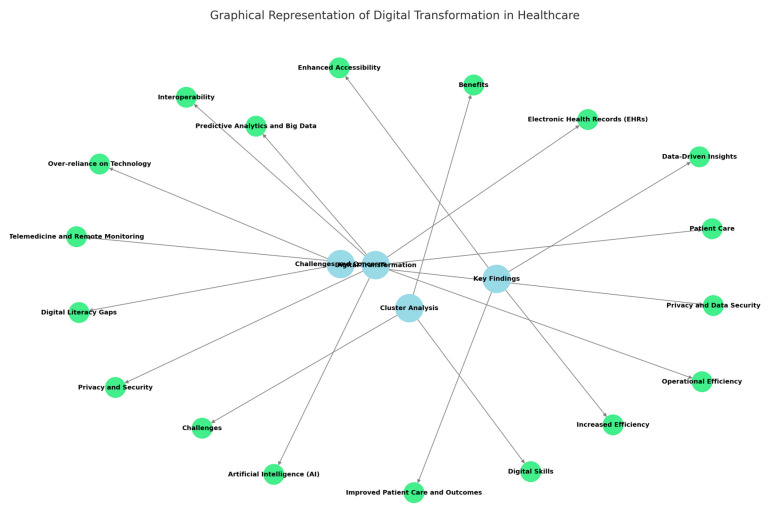
Major findings regarding digital transformation needs of health professionals obtained via written interviews.

We compared the findings of this questionnaire to the previous literature and existing research, such as studies emphasising the role of digital tools in improving patient care efficiency and patient outcomes (see Figure 3). However, our study uniquely identifies specific fears and concerns of healthcare professionals, such as job displacement and cost barriers, which are less explored in the previous literature.

## 6. Conclusions

The integration of digital health technologies into the healthcare industry holds immense potential to enhance efficiency, improve patient outcomes, and generate economic benefits. However, the widespread adoption of these technologies has been hindered by a lack of digital skills among healthcare professionals.

To bridge this digital skills gap, it is imperative that healthcare education and training programmes prioritise the development of digital proficiency. By equipping the next generation of healthcare providers with the necessary digital skills, organisations can streamline daily workflows, enhance decision-making, and ultimately provide more personalised and patient-centric care.

Digital skills are essential for health professionals to effectively navigate the complexities of modern healthcare delivery. The literature highlights the critical need for ongoing training and education to bridge the skills gaps, overcome barriers to adoption, and empower health professionals to harness digital technologies for enhanced patient care and innovation. As healthcare continues to advance in the digital age, acquiring and developing these skills will remain a vital priority for health professionals committed to providing high-quality, patient-centred care.

Moreover, the rapidly evolving healthcare landscape underscores the importance of establishing structured educational frameworks that integrate digital competencies into both foundational training and continuous professional development programmes. By equipping all healthcare workers with the necessary digital skills, it is possible to foster a more efficient, effective, and equitable healthcare system that addresses the diverse needs of patients across various contexts and settings. This will then enable healthcare professionals to leverage digital tools and technologies to streamline workflows, enhance clinical decision-making, and deliver more personalised and responsive care to patients.

Ultimately, the commitment to building digital literacy among health professionals will be a driving force in shaping a future in which technology and healthcare work hand in hand to deliver the best possible outcomes for all patients, ultimately improving patient experiences and health outcomes. As the healthcare industry continues to evolve, the acquisition and application of digital skills will be crucial for health professionals to remain at the forefront of innovation and provide the highest quality of care to the communities they serve.

## Figures and Tables

**Table 1:** Summary of the methods for identifying major digital skills in healthcare.

Method	Description	Examples
Analyse Industry Needs and Trends	Monitor technological advancements and healthcare digital transformation initiatives.	Telehealth adoption during the pandemic identified telehealth competency as critical.
Consult Healthcare Professionals and Experts	Conduct surveys, interviews, and focus groups to gather insights from practitioners on necessary digital skills.	Radiologists may highlight AI-based diagnostic tools as essential.
Examine Job Market and Role Requirements	Analyse job listings and professional certifications to identify in-demand digital skills.	EHR proficiency often required in healthcare job postings.
Look at Healthcare System Needs	Focus on skills that improve patient care efficiency, reduce errors, and ensure data security and privacy.	Predictive analytics for managing population health highlights the need for data analytics skills.
Incorporate Regulatory and Ethical Considerations	Ensure that digital skills align with compliance (e.g., HIPAA, GDPR) and ethical use of technology in healthcare.	Health data privacy laws emphasise the importance of data security skills.
Identify Gaps Through Competency Frameworks	Use digital competency frameworks (e.g., HIMSS) and self-assessment tools to identify skill gaps.	HIMSS framework highlights health informatics and telehealth as key digital skills.
Study Case Studies and Best Practices	Review successful case studies in which digital tools improved healthcare outcomes or operational efficiency.	Hospitals using AI for predictive analytics reveal the importance of AI skills.
Feedback from Continuous Learning and Development	Track trends in professional development, training programmes, and certifications for emerging digital skills.	Rising demand for AI and machine learning training suggests these skills are essential.

**Table 2:** Major digital skills in healthcare extracted from consulting health reports and scientific papers.

Skill	Description	Key Benefits
Electronic Health Record (EHR) Proficiency	Mastery of EHR systems for managing patient data, treatment plans, and care coordination.	Enhances care quality, safety, and cost-effectiveness through advanced features such as predictive analytics.
Telehealth Competency	Ability to conduct virtual consultations, manage remote monitoring, and engage patients using digital platforms.	Expands access to care, especially for underserved areas, improves patient satisfaction and equity.
Health Data Analysis	Proficiency in analysing health data to inform decisions on diagnosis, treatment, and preventive care strategies.	Enables personalised care, reduces medical errors, and drives innovation through actionable insights.
Digital Communication Tools Mastery	Effective use of digital tools (e.g., messaging platforms, video conferencing) for healthcare team coordination.	Improves real-time consultations, care coordination, and resource allocation, enhancing patient outcomes.
Adapting to Emerging Technologies	Staying up to date with AI, ML, robotics, VR, and other emerging technologies in healthcare.	Boosts workflow efficiency, improves decision-making, and enhances patient engagement and system responsiveness.

**Table 3:** Major digital skills from health professionals’ written statements about digital health and their needs.

Digital Skill	Description	Digital Tools
Electronic Health Record (EHR) Proficiency	Certification from HIMSS or EHR vendors (e.g., Epic, Cerner) Documented EHR usage (accurate data entry, retrieval) Audit trails of EHR usage Peer reviews on EHR efficiency and accuracy	Automatic writing (voice2text) Extract information from EHR
Telehealth Competency	Telehealth certification (e.g., ATA) Data on telehealth usage (consultation numbers, patient satisfaction) Recorded virtual consultation sessions Patient/peer feedback on telehealth services	Platforms for Telehealth
Health Data Analysis	Data-driven projects demonstrating improved care Case studies or reports on data-driven healthcare decisions Certification in health data analytics	Dashboard tools Health data analytics software
Adapting to Emerging Technologies	Hands-on use of emerging technologies (AI, ML, VR, robotics) Participation in tech-driven healthcare projects Certification in AI/ML, VR, or robotics in healthcare Blockchain	Specify tools for VR

**Table 4:** Barriers to digital skill adoption among health professionals.

Barrier	Description
Lack of Training and Education	Many health professionals receive limited formal training in digital skills during their education and professional development. This skills gap hinders their ability to effectively use digital tools and technologies, leaving them ill-equipped to navigate the increasingly digital landscape of healthcare.
Resistance to Change	Some health professionals may be hesitant to adopt new technologies due to concerns about workflow disruptions, data security, or the perceived complexity of learning new systems. This reluctance to embrace digital innovations can slow the pace of technological advancements in the healthcare sector.
Limited Resources	Access to technology and training opportunities may be restricted, particularly in resource-constrained settings such as underserved communities and rural areas. This disparity in access can exacerbate existing inequities in healthcare delivery.
Time Constraints	Health professionals often face heavy workloads, leaving little time for training and professional development in digital skills. The additional burden of acquiring new competencies may be perceived as an obstacle, making it challenging to prioritise digital skill development.

**Table 5:** Strategies for promoting digital skill adoption, as extracted from the literature review.

Strategy	Description
Comprehensive Training Programmes	Develop and implement targeted training initiatives that focus on enhancing digital proficiency, ensuring that health professionals can effectively use current digital tools and adapt to future technological advancements.
Continuous Professional Development	Encourage ongoing education and skill refinement through workshops, seminars, and online courses that keep health professionals updated on emerging digital health technologies and best practices.
Organisational Support and Infrastructure	Establish supportive infrastructures within healthcare organisations that facilitate access to necessary technologies and resources while promoting a culture that values and supports digital literacy and innovation.
Flexible Scheduling and Time Allocation	Provide health professionals with dedicated time and opportunities within their schedules to engage in digital skill development without adding to their existing workload pressures.
Addressing Equity in Access	Ensure equitable distribution of resources and training opportunities across all healthcare settings, including underserved and rural areas, to reduce disparities in digital skill adoption and healthcare delivery.

**Table 6:** Strategies for enhancing digital skills in healthcare.

Strategy	Description
Integrating Digital Skills Training into Healthcare Education	Incorporate comprehensive digital skills training into the curricula of healthcare education programmes (e.g., medical schools, nursing programmes, and allied health courses). This foundational training ensures that future health professionals are equipped with the necessary competencies to navigate the digital landscape, effectively using EHRs, telehealth platforms, data analytics tools, and other digital technologies integral to modern healthcare delivery.
Providing Ongoing Professional Development	Offer a range of continuing education programmes, workshops, and training opportunities to help health professionals stay current with the latest digital tools and technologies. These initiatives should enhance both technical and practical skills, empowering professionals to integrate new digital solutions into their daily workflows and optimise patient care.
Creating a Supportive Organisational Culture	Foster a culture within healthcare organisations that actively encourages and supports the adoption of digital technologies. This includes providing necessary resources, such as access to hardware, software, and IT support, creating dedicated training programmes, and incentivising the use of digital tools. Such a culture facilitates the seamless integration of technology into operations, thereby empowering health professionals to leverage these tools to their full potential.
Leveraging Technology to Enhance Training	Utilise innovative and technology-enabling learning platforms, such as online courses, virtual simulations, and interactive multimedia, to provide health professionals with flexible and accessible opportunities to develop their digital skills. These technology-enhanced learning modalities complement traditional in-person training, offering a more dynamic and engaging learning experience tailored to the needs and preferences of diverse health professionals.
Integrating Digital Skills Training into Healthcare Education	Incorporate comprehensive digital skill training into the curricula of healthcare education programmes (e.g., medical schools, nursing programmes, and allied health disciplines). This foundational training ensures that future health professionals are equipped with the necessary competencies to navigate the digital landscape, effectively utilising EHRs, telehealth platforms, data analytics tools, and other digital technologies integral to modern healthcare delivery.

## Data Availability

Data are available upon request after signing an NDA.
